# An optical and electrical study of full thermally activated delayed fluorescent white organic light-emitting diodes

**DOI:** 10.1038/s41598-017-06568-3

**Published:** 2017-07-24

**Authors:** Daniel de Sa Pereira, Paloma L. dos Santos, Jonathan S. Ward, Przemyslaw Data, Masato Okazaki, Youhei Takeda, Satoshi Minakata, Martin R. Bryce, Andrew P. Monkman

**Affiliations:** 1 Physics Department, Durham University, South Road, Durham, DH1 3LE United Kingdom; 2 Department of Chemistry, Durham University, South Road, Durham, DH1 3LE United Kingdom; 3 Centre of Polymer and Carbon Materials, Polish Academy of Science, M. Curie-Sklodowskiej 34, 41-819 Zabrze, Poland; 4 Faculty of Chemistry, Silesian University of Technology, M. Strzody 9, 44-100 Gliwice, Poland; 5 Department of Applied Chemistry Graduate School of Engineering Osaka University, Yamadaoka 2-1, Suita Osaka, 565-0871 Japan

## Abstract

We report on the engineering of full thermally activated delayed fluorescence – based white organic light emitting diodes (W-OLEDs) composed of three emitters (2,7-bis(9,9-dimethyl-acridin-10-yl)-9,9-dimethylthioxanthene-*S*,*S*-dioxide (**DDMA-TXO2**), 2,7-bis(phenoxazin-10-yl)-9,9-dimethylthioxanthene-*S*,*S*-dioxide (**DPO-TXO2**) and 3,11-di(10*H*-phenoxazin-10-yl)dibenzo[*a,j*]phenazine (**POZ-DBPHZ**) in two different hosts. By controlling the device design through the study of the emission of **DDMA-TXO2** and **DPO-TXO2**, the behaviour of **POZ-DBPHZ** in a device with more than one emitter, and the combination of the three materials, respectively, we show that external quantum efficiencies as high as 16% can be obtained for a structure with a correlated colour temperature close to warm white, together with colour rendering index close to 80. However it is in their performance stability that provides the true breakthrough: at 1000 cd/m^2^ the efficiencies were still above 10%, which is one of the best for this type of devices.

## Introduction

30 years of organic light-emitting diode (OLED) research, since Tang and VanSlyke’s^1^ first report, have transformed the optoelectronic landscape, especially within the smartphone and flat panel display industries^2^. This has given rise to remarkable OLED properties, which allow for light-weight devices with high efficiency, flexibility and low energy consumption using ultra-thin structures and tailored manufacturing.

A particular application that has shown great progress is solid state lighting (SSL) with white OLEDs (W-OLEDs), producing high white colour quality, homogeneous illumination, high quality colour rendering and stability^3^. With this in mind, multiple hybrid W-OLEDs implementing fluorescent^4^ or phosphorescent^5–7^ emitters, as well as exciplex^8^, excimer^9^, electroplex^10^, aggregated induced emission (AIE)^11, 12^ or quantum dots^13^ have been developed with various device structures.

When considering lighting systems, it is important to consider the effects of blue emitters in SSL devices^14^. Recent studies show that cooler lights (high illumination levels of blue light) can severely affect the secretion of melatonin, deregulating the circadian rhythm and increasing the eyes retinal light exposure, leading to higher phototoxicity levels that can have severe effects in cellular structures, test animals, and human fetal retinas^15, 16^. With increased use of SSL, it becomes imperative to develop white-light systems with lower contributions from blue emitters, so to produce warmer toned lighting and improve the quality of life.

A recent topic in the OLED lighting technology addresses the materials used as emitters and the strategies by which the 25% internal quantum efficiency (IQE) (dictated by the 1:3 ratio of singlet and triplet excitons) can be overcome, without the use of heavy metals like iridium or platinum. In 2012, Adachi and co-workers reported the use of purely fluorescent complexes with a small singlet-triplet (ΔE_ST_) energy splitting to promote efficient reverse intersystem crossing (rISC) and mediate the spin-flip back to the singlet state and 100% singlet harvesting in a thermally activated delayed fluorescence (TADF) process^17–19^. This results in two different emissions from the singlet state: in the ns range called prompt fluorescence (PF) and, typically in the µs range, delayed fluorescence (DF) *via* the reverse intersystem crossing (rISC) from the triplet to the singlet states^20–23^.

In regards to W-OLEDs, devices with maximum external quantum efficiencies (ƞ_ext_) above 20% have already been reported^5, 6^ using heavy, non-environmentally friendly and scarce materials while their complicated device structures imply increased production costs. Additionally, a few papers have reported white emission from both hybrid^24, 25^ and full TADF^26–30^ structures, with one of the latter devices reporting a maximum efficiency of 19%^30^ taking advantage of these materials’ high efficiencies and broad CT emission. Still, the challenge remains to find suitable TADF emitters and host materials that combine relatively low roll-off and high efficiency with stable optical and electrical properties, particularly for blue and red TADF materials. It is therefore crucial to design structures that promote high carrier balance inside each emitter, avoid quenching mechanisms^24^ and have a low contribution of blue emitters without affecting the white colour quality.

In this work, we have optimised a three-component, full TADF W-OLED structure considering optical applications in terms of commission internationale de l'éclairage (CIE 1931), correlated colour temperature (CCT) and colour rendering index (CRI) and electrical performances. Three TADF compounds previously studied by our group were chosen due to their exceptional performance, both in terms of photophysics and in devices– 2,7-bis(9,9-dimethyl-acridin-10-yl)-9,9-dimethylthioxanthene-*S*,*S*-dioxide (**DDMA-TXO2**)^31^, 2,7-bis(phenoxazin-10-yl)-9,9-dimethylthioxanthene-*S*,*S*-dioxide (**DPO-TXO2**)^32^ and 3,11-di(10*H*-phenoxazin-10-yl)dibenzo[*a,j*]phenazine (**POZ-DBPHZ**)^33^ as blue, green and orange emitters, respectively. After optimisation, efficiencies as high as 16%, 32.2 cd/A and 22.8  lm/W together with CRI of 77.6 and CCT of approximately 6200 K were obtained. More interestingly, at 1000 cd/m^2^, the efficiency remained above 10%, a promising low roll-off. This places the structure among the highest reported for TADF emitters, strongly suggesting that, by using standard transport materials and hosts one can easily obtain high efficient and stable W-OLEDs with TADF emitters.

## Results

**Figure 1:**
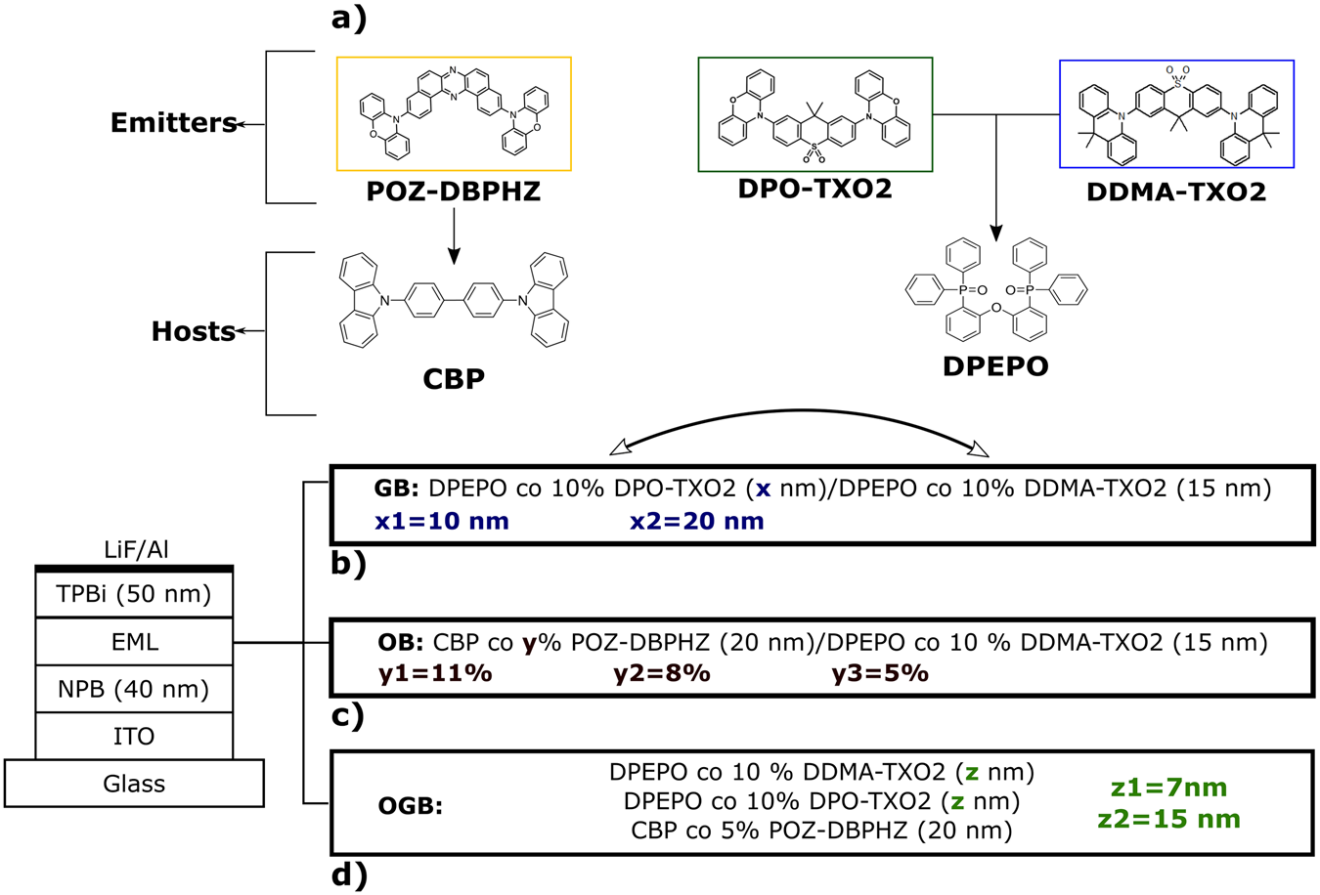
(**a**) Molecular structures of host and guest molecules used as emitting layers in a general device structure (Tables S1–3). Three studies were conducted: (**b**) one with green and blue emitters with thickness (x) of the green’s Host-Guest system between 10 and 20 nm, (**c**) another with orange (concentrations – y – of 5, 8 and 11%) and blue. The best results were merged into the final device structure (**d**) and thicknesses of DPEPO (z) were varied between 7 and 15 nm.

To understand the dynamics inside this TADF white OLED with single emitting layers, three sets of studies were conducted, culminating in the optimised white structure. Both electrical and optical aspects were considered in this optimisation study. The molecular structure of each TADF emitter and corresponding host are shown in Fig. 1a. For more details on each emitter, host and energy levels, see Tables S1–3 and Figure S1 in the supporting information (SI) section.

### Green-blue Structure

**Figure 2:**
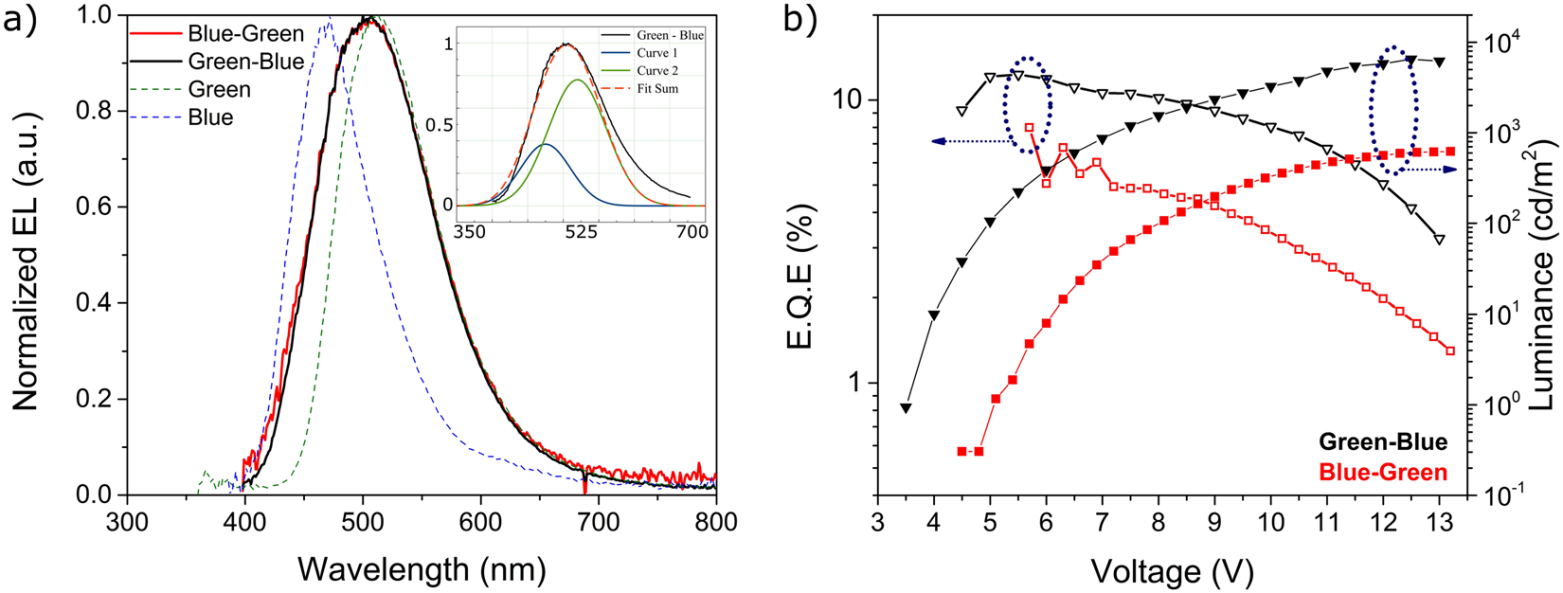
Study of the orientation of the layers in a green-blue structure. (**a**) Electroluminescence (EL) spectra of both devices together with the EL of each material independently, taken from^31, 32^. The inset shows the deconvolution of the EL spectrum of the green-blue structure. (**b**) Electrical performance of the devices used for both structures.

The first set (Fig. 1b) focused on the positioning and thickness of the DPEPO layers of the Blue-Green complex. For the first part, two devices with blue-green and green-blue orientations were deposited with fixed thicknesses of 15 nm for both blends. The results are shown in Fig. 2.

From Fig. 2a it is clear that the order of green and blue within the device has no effect in its emission properties as both showed similar electroluminescence (EL) spectra. Rather than having individualized peaks, both structures showed an emission that is the sum of the independent emitters. This can be supported by the overlap of the EL spectra with both green and blue individual devices. The inset of Fig. 2a shows its deconvolution into two emitters to understand the contribution of each emitter. Evidently, given the overall green emission, the biggest contributor to the device performance is **DPO-TXO2** which represents around 70% of the contribution. The remaining 30% comes from **DDMA-TXO2**. This is a good indication of a potential Solid State Lighting (SSL) device with low emission from the blue counterpart, but enough contribution to obtain white OLEDs with further development. In terms of electrical performance (Fig. 2b), the green-blue structure showed the best set of results with lower turn-on voltage (V_on_), higher ƞ_ext_ and luminance. Also, the roll-off of green-blue was lower, indicating a more stable device. An explanation for this lies in the carrier balance inside both layers (Figure S2). DPEPO is a known electron transport material^34^. Its’ use in device production is based on the high band gap and high polarity even though the hole mobility is relatively slow. And because both structures have similar emissions, the better electrical performance is a result of a better electron-hole distribution in the green-blue structure. DDMA-TXO2 has lower HOMO than DPO-TXO2, therefore direct injection in green-blue is much more favourable than in the blue-green resulting in a lower turn-on voltage and a better carrier density without affecting the emission. Figure S3 shows the current efficiency and luminous efficacy to confirm that indeed the carrier balance plays a pivotal role. It is worth noting the ƞ_ext_ above 10% from the blue-green structure, especially at luminance values above 1000 cd/m^2^. This result prompted us to increase the green layer’s DPEPO thickness to 20 nm to improve, even further, this carrier balance. From Figure S4, the maximum ƞ_ext_ increased from approximately 12% to around 16%, maintaining the low roll-off and increasing the maximum luminance to around 8000 cd/m^2^ compared to the 6000 cd/m^2^ from the first green-blue structure. At around 8 V, there is a bump in the electrical performance which can be assigned to an increased injection of carriers to DPEPO. Still, the EL remained the same, as seen from the onset of Figure S4. Therefore, the use of thinner electron transport parts together with the sum of the individual emissions of green and blue counterparts, constitute the main results of this part of the study.

### Orange-blue Structure

**Figure 3:**
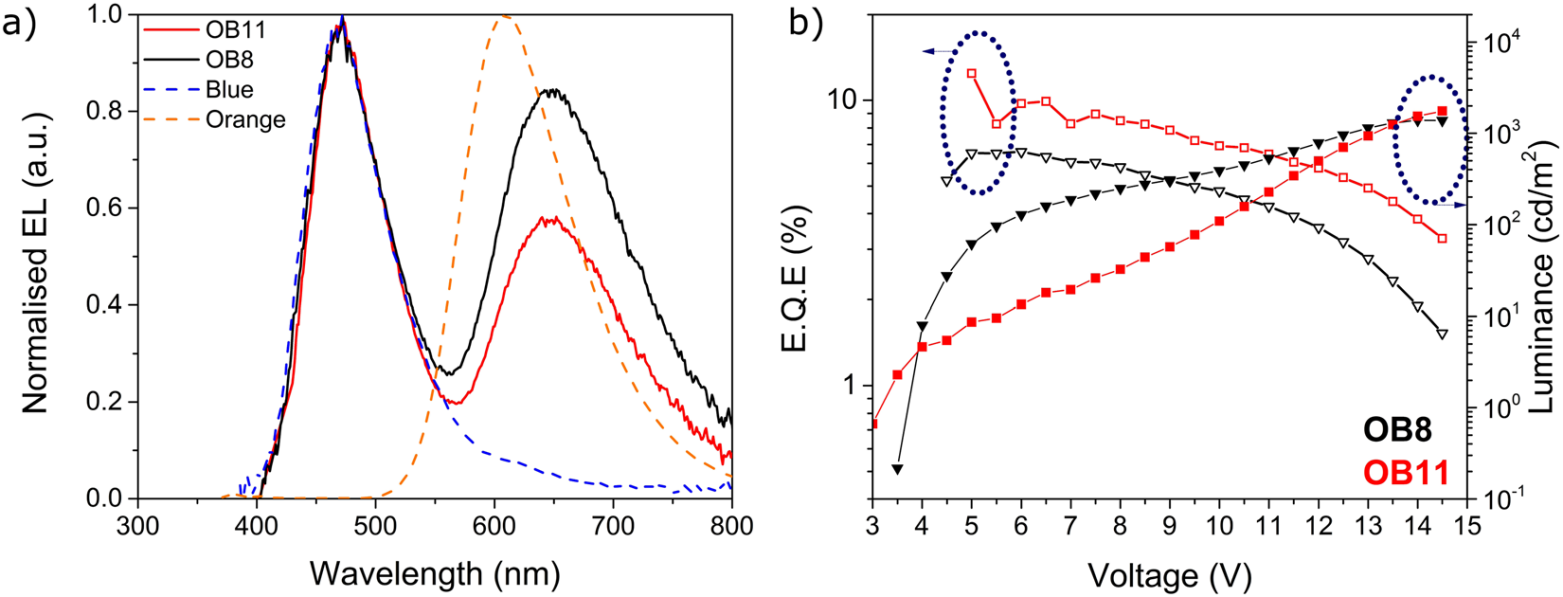
Study of the concentration of POZ-DBPHZ in an orange-blue structure with relative concentrations of orange of 8% and 11%. (**a**) Electroluminescence (EL) spectra of both devices together with the EL of each material independently, taken from^36, 37^. (**b**) Electrical performance of the devices used for both structures.

The second part of this study (Fig. 1c) focused on the interaction between the blue and orange TADF emitters. Fixing both hosts’ thicknesses (from their single emission structures) and **DDMA-TXO2**’s concentration, the concentration dependence of **POZ-DBPHZ** was varied between 8% and 11%; the results are shown in Fig. 3. From the EL spectra, two distinct features can be seen: one at 450 nm overlapping with the emission of **DDMA-TXO2** on its own and another at around 650 nm, red-shifted from the emission profile of **POZ-DBPHZ**, which we believe to be evidence of the appearance of an excimer which picks out the low energy excimer states on charge recombination the same way that keto defects in polyfluorene giving rise to different EL spectra to the PL^35^. This needs to be studied in more detail though it can be sustained by the relative decrease of this second feature when the concentration of **POZ-DBPHZ** was increased from 8 to 11%.

**Figure 4:**
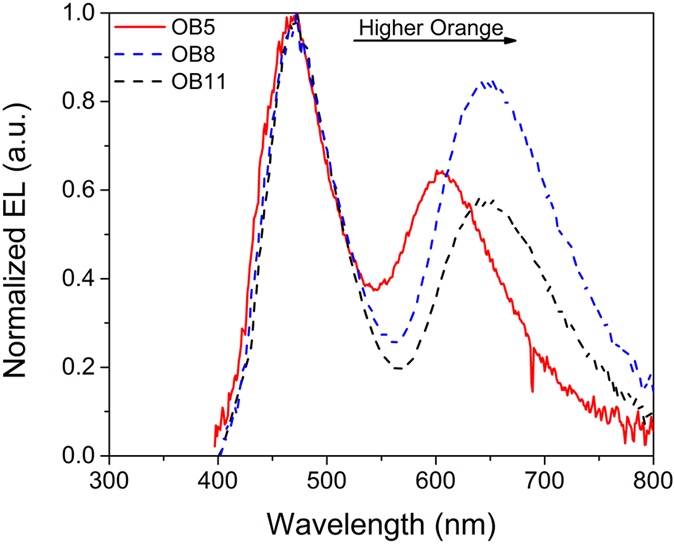
Orange-blue structure with relative concentrations of orange of 5% a relative comparison with OB7 and OB11.

To prove that an energy transfer process occurs from **DDMA-TXO2 (B)** or/and **DPO-TXO2 (G)** to **POZ-DBPHZ (O)**, the absorption and PL spectra of these three and combinations OB, GO and BGO were measured. The results are shown in Figure S5 strongly suggesting that the emission of **POZ-DBPHZ** can be enhanced in whatever matrix it is blended with. The concentration of **POZ-DBPHZ** was therefore decreased to 5% and another blue-orange device was obtained and its emission is shown in Fig. 4. **POZ-DBPHZ** emission reverted to 600 nm, hence sustaining the formation of the excimer.

The coverage of most of the entire visible spectrum, i.e. close to white, led us to study the optical characteristics of this structure. Table 1 together with Figure S6 shows the figures of merit of this study. Again, while both devices show colour coordinates around white, the absence of a green emitter resulted in a poor colour rendering ability. The decrease in **POZ-DBPHZ**’s emission shifted the colour temperature to cooler values, as seen by the increase of CCT while the higher emission of **DDMA-TXO2** (when compared with **POZ-DBPHZ**) in device OB11 increased slightly the CRI, phenomena typical of blue emitters. As expected, the blue-shift in the emission of **POZ-DBPHZ** considerably increased the CRI of device OB5 to 73.3. Using the optimised green-blue structure of the previous study and merging with the ratios studied here allowed us to combine both structures into the orange-green-blue device.

**Table 1:** Figures of merit of the devices used in the concentrations studies of the orange-blue structure.

Device	CIEx	CIEy	CRI	CCT (K)
OB5	0.31	0.28	73.3	7024.0
OB8	0.32	0.29	38.7	6109.3
OB11	0.28	0.26	40.0	11878.9

### Orange-green-blue structure

**Figure 5:**
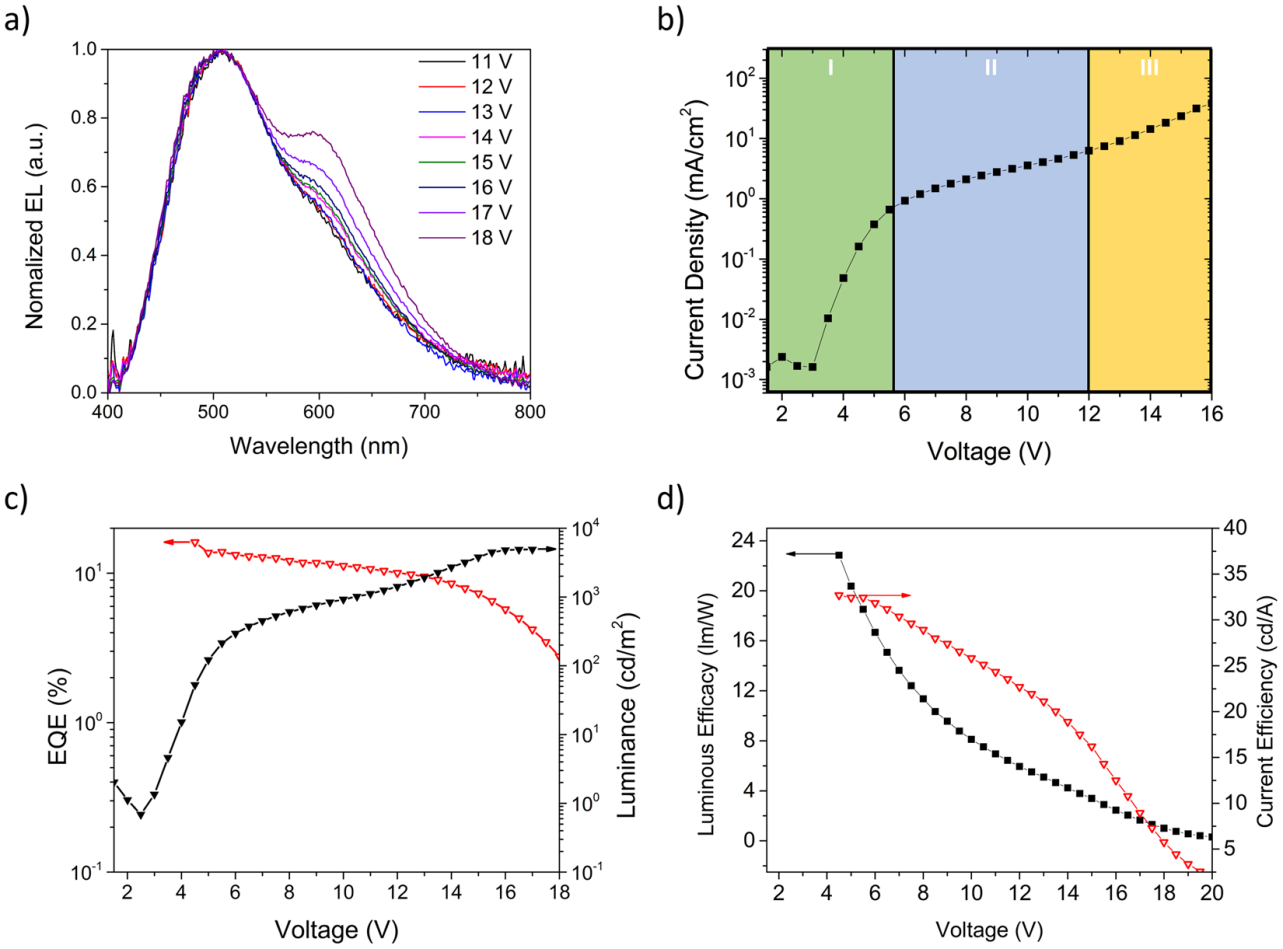
Orange-green-blue study of a device containing the optimised structure for a W-OLED. (**a**) EL spectra at different voltages. (**b**) Current density-voltage (JV) with three different regimes: I the space charge limited current (SCLC); II and III the different carrier balance regimes. (**c**) ƞ_ext_ and luminance dependences with voltage and (**d**) Luminous and current efficiencies of the device.

The information gained from the GB and OB devices were combined into a structure with the three emitters in three different layers (Fig. 1d). Besides providing an enhancement in the efficiency properties, **DPO-TXO2** was added to the previous OB structure to enhance the ƞ_ext_, to improve the rendering at visible wavelengths, i.e. an improved CRI and, above all, to decrease the contribution of **DDMA-TXO2** as a blue emitter. Also, the three main aspects from the previous sections were taken into consideration: 1. The decrease of energy barriers between consecutive emitters; 2. Thicker hole transport layers to improve carrier balance; 3. Lower concentrations of **POZ-DBPHZ** to avoid excimer formation. Subsequently, one final study was conducted with an orange-green-blue (OGB) structure, where the DPEPO layer thicknesses were changed between 15 nm (Fig. 5) and 7 nm (Figure S7) to see an effect on the electron injection to CBP. Although the thinner device, as expected showed a better emission from **POZ-DBPHZ**, its efficiency values also decreased, mainly due to the lower contribution of the high efficiency emitters. Figure 5a shows the emission of the W-OLED at different voltages. The decrease in **POZ-DBPHZ** concentration, together with the decrease in DPEPO thicknesses (from 7 to 15 nm), not only decreased the formation of excimers, resulting in emission from pure compound in the device, but also promoted a substantial increase in **POZ-DBPHZ**’s emission with increasing voltage. At higher voltages, there is a change in carrier balance regime where the carrier density increased the orange emission compared to the orange-blue complex. This can be seen in Fig. 5b where the space charge limited current (SCLC), i.e. the current is dominated by charge carriers injected from the contacts and so I~V^2^, in region I is followed by two distinct current behaviours (regions II and III). From Fig. 5b and c, the turn-on voltage of the device is 2.5 V and can reach brightness levels as high as 5000 cd/m^2^ (~16 V), a practical value for lighting applications^38^. More impressive is the ƞ_ext_ that can reach values of 16% and stabilize at around 13%. At 1000 cd/m^2^ ƞ_ext_ was still around 10% from which it starts to decrease, as expected given the potential stress upon the device. In terms of other efficiencies (Fig. 5d), the maximum current efficiency and power efficiency and external quantum efficiency were 31.2 cd/A and 23.6 lm/W, respectively, results comparable with the individual emitter devices. At 1000 cd/m^2^ the values are as high as 22.8 cd/A and 7.8 lm/W which is an indication of low roll-off. At this particular device structure, the efficiency values are among the highest reported for full TADF WOLEDs, making this structure promising for lighting applications pending further optimisations. Table 2 highlights all the electrical properties of this device and the values of each efficiency at 100 and 1000 cd/m^2^ showing its electrical stability. Figure S7 shows the same results for the thinner device and Table S4 summarizes both its electrical and optical properties.

**Table 2:** Electrical properties of the orange-green-blue device including luminance and efficiency values.

V_ON_ (V)	L_MAX_ (cd/m^2^)	${\eta }_{{ext}},{\max }$ (%)	${\eta }_{L},{\max }$ (cd/A)	${\eta }_{P},{\max }$ (lm/W)	${\eta }_{{ext}}$ ^1^ (%)	${\eta }_{L}$ ^1^ (cd/A)	${\eta }_{P}$ ^1^ (lm/W)	${\eta }_{{ext}}$ ^2^ (%)	${\eta }_{L}$ ^2^ (cd/A)	${\eta }_{P}$ ^2^ (lm/W)
2.5	4924	16.1	32.7	22.4	13.7	32.4	20.4	11.0	25.1	7.5

^1^Values taken at 100 cd/m^2^.

^2^Values taken at 1000 cd/m^2^.

Moving on to colour properties, Table 3 shows the change in figures of merit of the OGB device at different voltages. Below 14 V the values remain constant (Fig. 5a). The introduction of **DPO-TXO2** in the OB structure red-shifted the first peak and stabilized the colour coordinates and colour temperatures while increasing considerably the CRI. When the emission of **POZ-DBPHZ** increased, the CRI improved as a result of a better rendering of reddish colours. A similar effect can be seen in the thinner device (Figure S7) where the improved carrier density in **POZ-DBPHZ** red-shifted the CCT and increased the CRI of the devices, even though their electrical performance decreased. Therefore, we have tailored the optical and electrical properties of a TADF white structure with simple device modifications making these values comparable to similar light sources, currently available commercially (see Figure S8).

**Table 3:** Colour figures of merit of the orange-green-blue device at different voltages.

Voltage (V)	CIEx	CIEy	CCT (K)	CRI
14	0.30	0.40	6313	77.6
16	0.31	0.40	6145	79.9
18	0.33	0.39	5505	86.7

## Discussion

We successfully combined three different TADF emitters into a W-OLED structure, and the device’s optical and electrical characteristics were studied in detail. By applying three different structures, we observed that the dual emission in the final device comes from: 1 – the superposition of the blue and green emitter and a lower contribution from the blue emitter, an important aspect given the current lighting paradigm of the phototoxicity from blue emitters, and 2 – from the use of low concentrations of the orange emitter to avoid the formation of excimers. We also show that given the device structure, for a higher carrier density control, the low energy gap materials should be closer to the hole injection layers and their ratios are extremely important to secure good colour rendering properties. With simple device modifications, we can obtain different colour-efficient or electrical-efficient devices. From this optimisation, devices with efficiencies as high as 16% and CRI close to 80 can be obtained. Also, the ${\eta }_{{ext}}$ of 11%, even at luminance above 1000 cd/m^2^, is achieved which places it among the highest reported for full TADF W-OLEDs^26–30^. Based on the colour properties of such devices, their efficiencies and stabilities, we propose a structure which is among the best for lighting applications using purely TADF emitters, known transport materials and hosts, a key aspect when considering cost effective structures. With use of a higher ${\eta }_{{ext}}$ green emitter, we believe even higher electrical performances and better optical properties can be achieved. The further introduction of another red compound could further decrease the CCT and improve the CRI.

## Methods

The OLEDs from this study were fabricated on patterned indium-tin-oxide (ITO) coated glass with sheet resistance of 20 Ω/sq and ITO thickness of 100 nm. After loading the pre-cleaned substrates into a Kurt J. Lesker Spectros II deposition chamber, both the small molecules and the cathode layers were thermally evaporated with a pressure of no more than 10^−6^ mbar for a pixel size of 16 and 8 mm^2^. For each, *N*,*N*′-di(1-naphthyl)-*N*,*N*′-diphenyl-(1,1′-biphenyl)-4,4′-diamine (**NPB**), 2,2′,2″-(1,3,5-benzinetriyl)-tris(1-phenyl-1*H*-benzimidazole) (**TPBi**) and lithium fluoride (**LiF**) were used as hole transport layer (HTL), electron blocking layer (EBL), hole blocking layer (HBL), electron transport layer (ETL) and electron injection layer (EIL), respectively. Aluminium was used as cathode. Finally, as host material for each emitting layer, 4,4′-bis(*N*-carbazolyl)-1,1′-biphenyl (**CBP**) was used for **POZ-DBPHZ** and bis[2-(diphenylphosphino)phenyl] ether oxide (**DPEPO**) for both **DDMA-TXO2** and **DPO-TXO2**. NPB, TPBi, DPEPO and CBP were purchased from Sigma Aldrich.

For absorption and photoluminescence (PL) studies, toluene solutions of each emitter of 3 mM were prepared, and around 50 and 90 µL were drop-casted, respectively. The resulting samples were left under vacuum overnight to remove any left solvent. All the solutions were diluted and stirred for no less than two hours to ensure good solution homogeneity. Absorption and emission spectra were collected using a UV-3600 double beam spectrophotometer (Shimadzu), and a Fluorolog fluorescence spectrometer (Jobin Yvon).

## Electronic supplementary material

Revised SI

## References

[1] Tang CW, VanSlyke SA (1987). Organic electroluminescent diodes. Appl. Phys. Lett..

[2] Tremblay, J.-F. The rise of OLED displays. *C&EN* (2016). Available at: http://cen.acs.org/articles/94/i28/rise-OLED-displays.html. (Accessed: 1st December 2016).

[3] Reineke S, Thomschke M, Lüssem B, Leo K (2013). White organic light-emitting diodes: Status and perspective. Rev. Mod. Phys..

[4] Pereira D, Pinto A, California A, Gomes J, Pereira L (2016). Control of a White Organic Light Emitting Diode’s emission parameters using a single doped RGB active layer. Mater. Sci. Eng. B.

[5] Xue MM (2016). De Novo Design of Boron-Based Host Materials for Highly Efficient Blue and White Phosphorescent OLEDs with Low Efficiency Roll-Off. ACS Appl. Mater. Interfaces.

[6] Udagawa K, Sasabe H, Igarashi F, Kido J (2016). Simultaneous Realization of High EQE of 30%, Low Drive Voltage, and Low Efficiency Roll-Off at High Brightness in Blue Phosphorescent OLEDs. Adv. Opt. Mater..

[7] Du M (2016). Novel Emitting System Based on a Multifunctional Bipolar Phosphor: An Effective Approach for Highly Efficient Warm-White Light-Emitting Devices with High Color-Rendering Index at High Luminance. Adv. Mater..

[8] Hung W-Y (2014). The First Tandem, All-exciplex-based WOLED. Sci. Rep..

[9] Baryshnikov GV (2016). Nine-ring angular fused biscarbazoloanthracene displaying a solid state based excimer emission suitable for OLED application. J. Mater. Chem. C.

[10] Chen FP (2010). White organic light-emitting diodes based on electroplex from polyvinyl carbazole and carbazole oligomers blends. Chinese Phys. B.

[11] Mo HW (2016). Color Tuning of Avobenzone Boron Difluoride as an Emitter to Achieve Full-Color Emission. Adv. Funct. Mater..

[12] Lee Y-T, Chang Y-T, Chen C-T, Chen C-T (2016). The first aggregation-induced emission fluorophore as a solution processed host material in hybrid white organic light-emitting diodes. J. Mater. Chem. C.

[13] Ryu SY (2016). Optical efficiency enhancement in white organic light-emitting diode display with high color gamut using patterned quantum dot film and long pass filter and fluorescence Optical efficiency enhancement in white organic light-emitting diode display with h. Jpn. J. Appl. Phys..

[14] Jaadene I (2015). Retinal damage induced by commercial light emitting Diodes (LED). Free Radic. Biol. Med..

[15] Duffy JF, Czeisler CA (2009). Effect of Light on Human Circadian Physiology. Sleep. Med. Clin..

[16] Behar-Cohen F (2011). Light-emitting diodes (LED) for domestic lighting: Any risks for the eye?. Prog. Retin. Eye Res..

[17] Wilkinson, F. & Horrocks, A. R. in *Luminescence in Chemistry* 116–153 (Van Nostrand, 1968).

[18] Uoyama H, Goushi K, Shizu K, Nomura H, Adachi C (2012). Highly efficient organic light-emitting diodes from delayed fluorescence. Nature.

[19] Gibson J, Monkman AP, Penfold TJ (2016). The Importance of Vibronic Coupling for Efficient Reverse Intersystem Crossing in Thermally Activated Delayed Fluorescence Molecules. Chem. Phys. Chem.

[20] Graves D, Jankus V, Dias FB, Monkman A (2014). Photophysical investigation of the thermally activated delayed emission from films of m-MTDATA:PBD exciplex. Adv. Funct. Mater..

[21] Ward JS (2016). The interplay of thermally activated delayed fluorescence (TADF) and room temperature organic phosphorescence in sterically-constrained donor–acceptor charge-transfer molecules. Chem. Commun..

[22] Dias FB (2016). The Role of Local Triplet Excited States in Thermally-Activated Delayed Fluorescence: Photophysics and Devices. Adv. Sci..

[23] Etherington MK (2016). Revealing the spin–vibronic coupling mechanism of thermally activated delayed fluorescence. Nat. Commun..

[24] Wu Z (2016). Management of Singlet and Triplet Excitons: A Universal Approach to High-Efficiency All Fluorescent WOLEDs with Reduced Efficiency Roll-Off Using a Conventional Fluorescent Emitter. Adv. Opt. Mater..

[25] Lee SE (2016). Efficient triplet harvesting of hybrid white organic light-emitting diodes using thermally activated delayed fluorescence green emitter. J. Photonics Energy.

[26] Nishide J, Nakanotani H, Hiraga Y, Adachi C (2014). High-efficiency white organic light-emitting diodes using thermally activated delayed fluorescence. Appl. Phys. Lett..

[27] Duan C (2016). Multi-dipolar Chromophores Featuring Phosphine Oxide as Joint Acceptor: A New Strategy toward High-Efficiency Blue Thermally Activated Delayed Fluorescence Dyes. Chem. Mater..

[28] Liu Z (2017). Simple-structure organic light emitting diodes: Exploring the use of thermally activated delayed fluorescence host and guest materials. Org. Electron..

[29] Yu Y (2014). Fluorinated 9,9[prime or minute]-bianthracene derivatives with twisted intramolecular charge-transfer excited states as blue host materials for high-performance fluorescent electroluminescence. J. Mater. Chem. C.

[30] Liu W (2016). High Performance All Fluorescence White Organic Light Emitting Devices with A Highly Simplified Structure Based On Thermally Activated Delayed Fluorescence Dopants and Host. ACS Appl. Mater. Interfaces.

[31] Dos Santos PL, Ward JS, Bryce MR, Monkman AP (2016). Using Guest-Host Interactions to Optimize the Efficiency of TADF OLEDs. J. Phys. Chem. Lett..

[32] Dos Santos PL (2016). Engineering the singlet-triplet energy splitting in a TADF molecule. J. Mater. Chem. C.

[33] Data P (2016). Dibenzo[a,j]phenazine-Cored Donor-Acceptor-Donor Compounds as Green-to-Red/NIR Thermally Activated Delayed Fluorescence Organic Light Emitters. Angew. Chem., Int. Ed..

[34] Xu H (2006). Application of chelate phosphine oxide ligand in EuIII complex with mezzo triplet energy level, highly efficient photoluminescent, and electroluminescent performances. J. Phys. Chem. B.

[35] List EJW, Guentner R, Scanducci de Freitas P, Scherf U (2002). The Effect of Keto Defect Sites on the Emission Properties of Polyfluorene-Type Materials. Adv. Mater..

[36] Dos Santos PL, Dias FB, Monkman AP (2016). An Investigation of the Mechanisms Giving Rise to TADF in Exciplex States. J. Phys. Chem. C.

[37] Data P (2016). Exciplex Enhancement as a Tool to Increase OLED Device Efficiency. J. Phys. Chem. C.

[38] Gather MC, Köhnen A, Meerholz K (2011). White organic light-emitting diodes. Adv. Mater..

