# Supplementary material for: An optical and electrical study of full thermally activated delayed fluorescent white organic light-emitting diodes
Source: Sci Rep. 2017 Jul 24;7:6234. doi: 10.1038/s41598-017-06568-3 (PMC5524785; doi:10.1038/s41598-017-06568-3)
Supplement: Supplementary file 1 — Revised SI [file 41598_2017_6568_MOESM1_ESM.pdf]

Supplementary Information

An optical and electrical study of full thermally activated delayed fluorescent white organic light-emitting diodes

Daniel de Sa Pereira[1]*, Paloma L. dos Santos[1], Jonathan S. Ward[2], Przemyslaw Data[1,3,4], Masato Okazaki[5], Youhei Takeda[5], Satoshi Minakata[5], Martin R. Bryce[2] and Andrew P. Monkman[1]

[1] Physics Department, Durham University, South Road, Durham DH1 3LE, United Kingdom.

[2] Department of Chemistry, Durham University, South Road, Durham DH1 3LE, United Kingdom.

[3] Centre of Polymer and Carbon Materials, Polish Academy of Science, M. Curie-Sklodowskiej 34, 41-819 Zabrze, Poland.

[4] Faculty of Chemistry, Silesian University of Technology, M. Strzody 9, 44-100 Gliwice, Poland.

[5] Department of Applied Chemistry Graduate School of Engineering Osaka University, Yamadaoka 2-1, Suita Osaka 565-0871, Japan.

daniel.a.pereira@durham.ac.uk

p.l.dos-santos@durham.ac.uk

jonathan.ward@durham.ac.uk

przemyslaw.data@durham.ac.uk

m-okazaki@chem.eng.osaka-u.ac.jp

takeda@chem.eng.osaka-u.ac.jp

minakata@chem.eng.osaka-u.ac.jp

m.r.bryce@durham.ac.uk

a.p.monkman@durham.ac.uk

Corresponding Author:

Daniel A. de Sa Pereira

E-mail: daniel.a.pereira@durham.ac.uk

Tel: +44 (0) 7764510766

Table S1. Electrical properties of the devices evaporated using the TADF emitters as single emitting devices. All data was collected from each article, available online. [1–3]

	Ref.	Host	HOMO (eV)	LUMO (eV)	Triplet level (eV)[1]	λ max (nm)	V_{ON} (V)	L max (cd/m²)	η_{ext}, max (%)	η_{L}, max (cd/A)	η_{P}, max (lm/W)	η_{ext} (%)[2]	η_{L} (cd/A)[2]	η_{P} (lm/W)[2]	$L_{90\%}$ (cd/m²)	CIE (x,y)
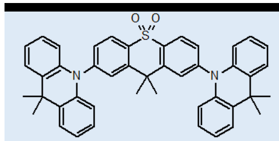 DDMA–TXO2	1	DPEPO	−5.80	−2.40	3.02	465	3.5	>2.500	22.4	NR	17.2	18	NR	NR	350	(0.16,0.24)
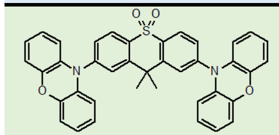 DPO–TXO2	2	DPEPO	−5.37	−2.61	2.83	520	3.5	>10.000	13.5	28.1	35.7	11.5	32	20	500	(0.26,0.51)
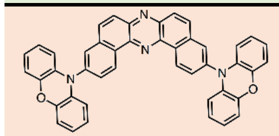 POZ-DBPHZ	3	CBP	−5.36	−3.38	2.21	600	3.7	>30.000	16	>30	NR	14	>30	NR	>2000	NR

Table S2. Energy levels of both hosts used in this study. [4,5]

HOST		HOMO (eV)	LUMO (eV)	Triplet level (eV)
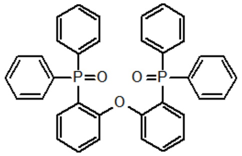	DPEPO	−6.1	−2.0	2.99[4]
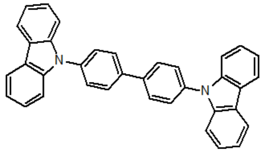	CBP	−6.0	−2.9	2.56[5]

Table S3. Molecular structures, the energy levels of each commercially available material and their function in the devices used in this study.

Compound		Function	HOMO (eV)	LUMO (eV)	Triplet level (eV)
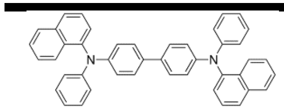	NPB	HTL	−5.5	−2.4	2.3
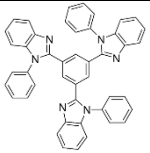	TPBi	ETL	−6.2	−2.7	2.7

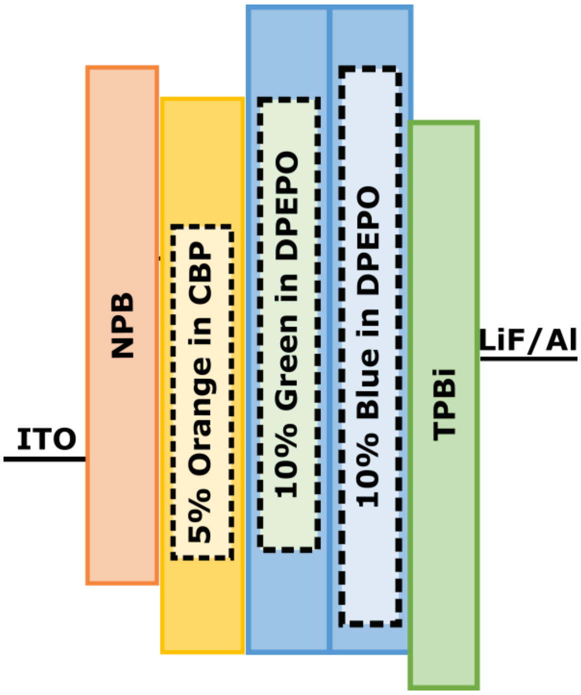

Figure S1. Energy Levels of the final white device.

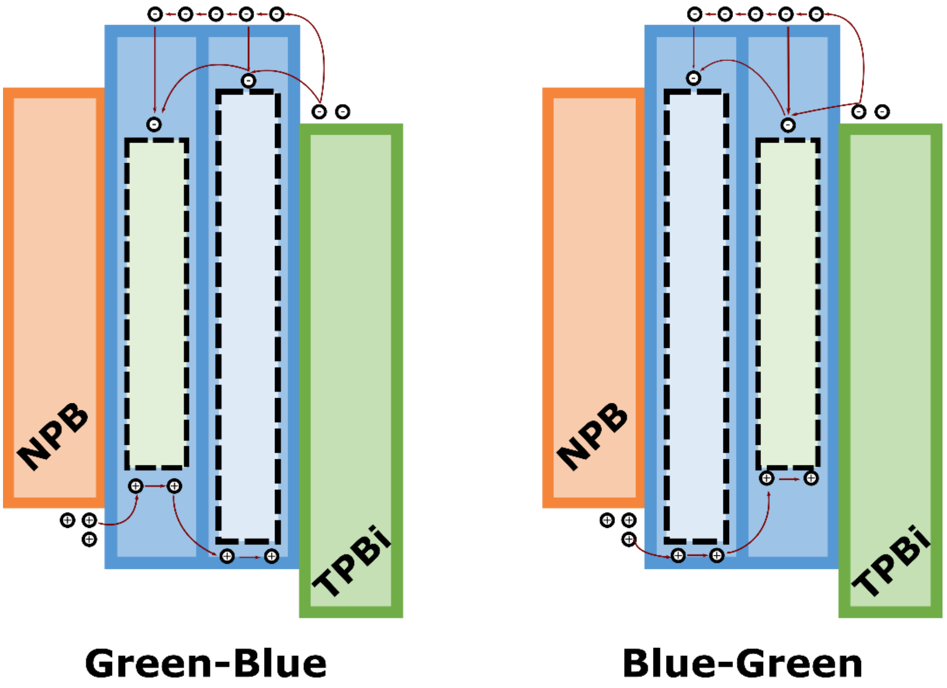

Figure S2. Direct hole injection mechanism in green-blue and blue-green structures. The direct injection from **NPB** to **DPO-TXO2** is vastly enhanced by the no-energy gap in the green-blue structure.

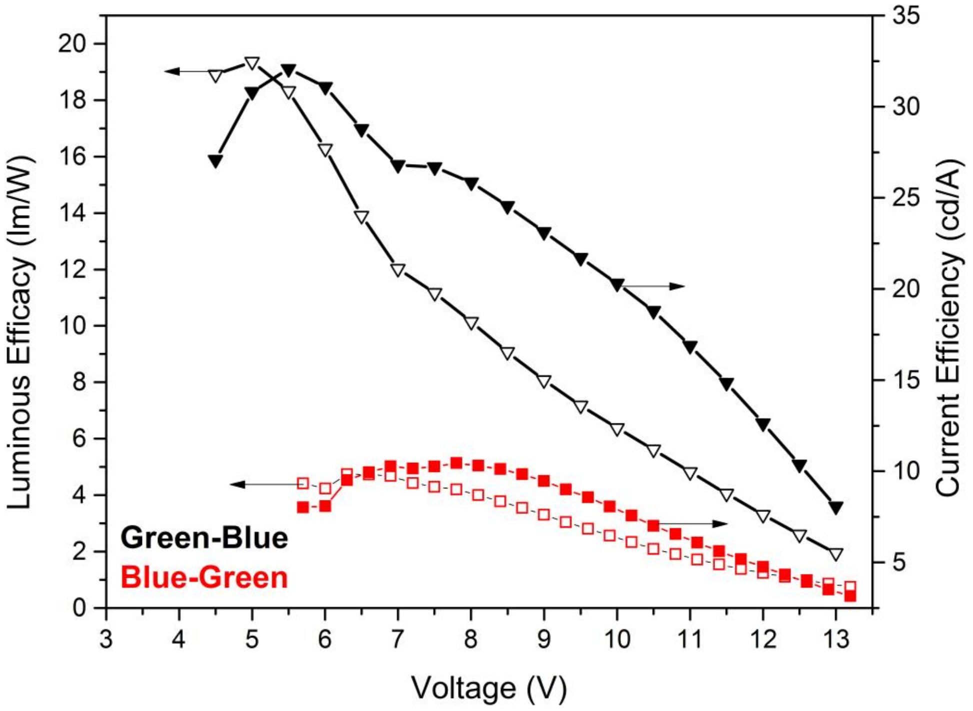

Figure S3. The luminous efficacy and the current efficiency of the devices green-blue and blue-green. Blue-green has much smaller efficiency values as the carrier balance in the structure pays a pivotal role in the overall operating mechanism.

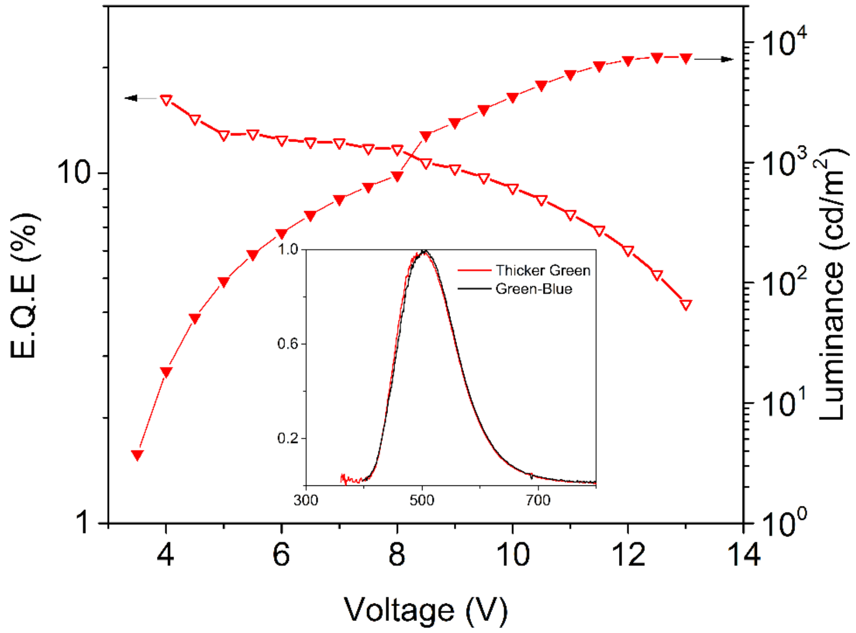

Figure S4. Electrical performance of the Green-blue device with thicker layer of green. The inset shows the normalized EL spectrum overlapped with the device from the first part of this study.

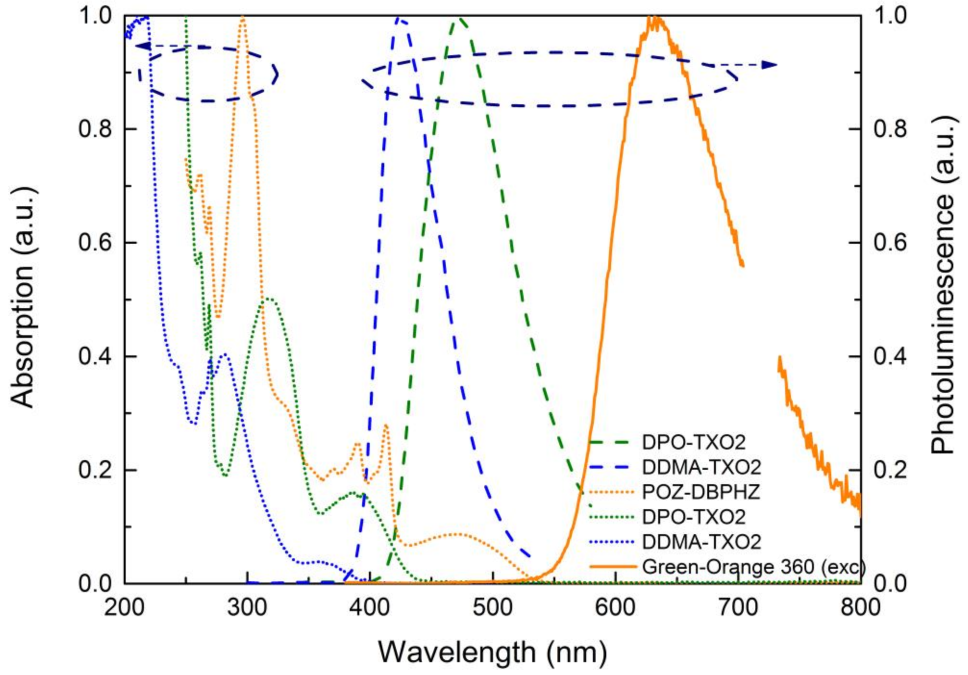

Figure S5. The absorption of POZ-DBPHZ, DPO-TXO2, DDMA-TXO2 together with the photoluminescence of DPO-TXO2 and DDMA-TXO2 and a blend of DPO-TXO2 and POZ-DPHZ excited at 360 nm. It shows that, by exciting the blend, only emission from POZ-DBPHZ is seen, confirming the efficient energy transfer.

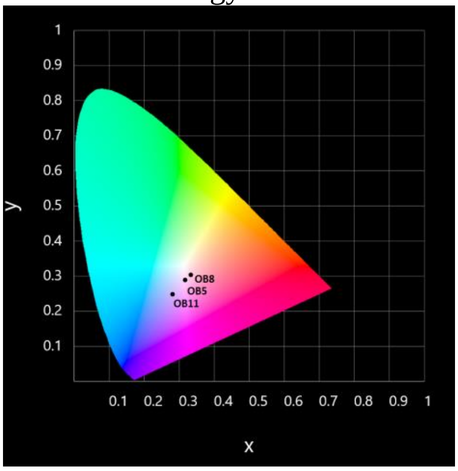

Figure S6. The CIE 1931 coordinates of devices OB5, 8 and 11 showing both the effects of the concentration of POZ-DBPHZ and the formation of aggregates in the device.

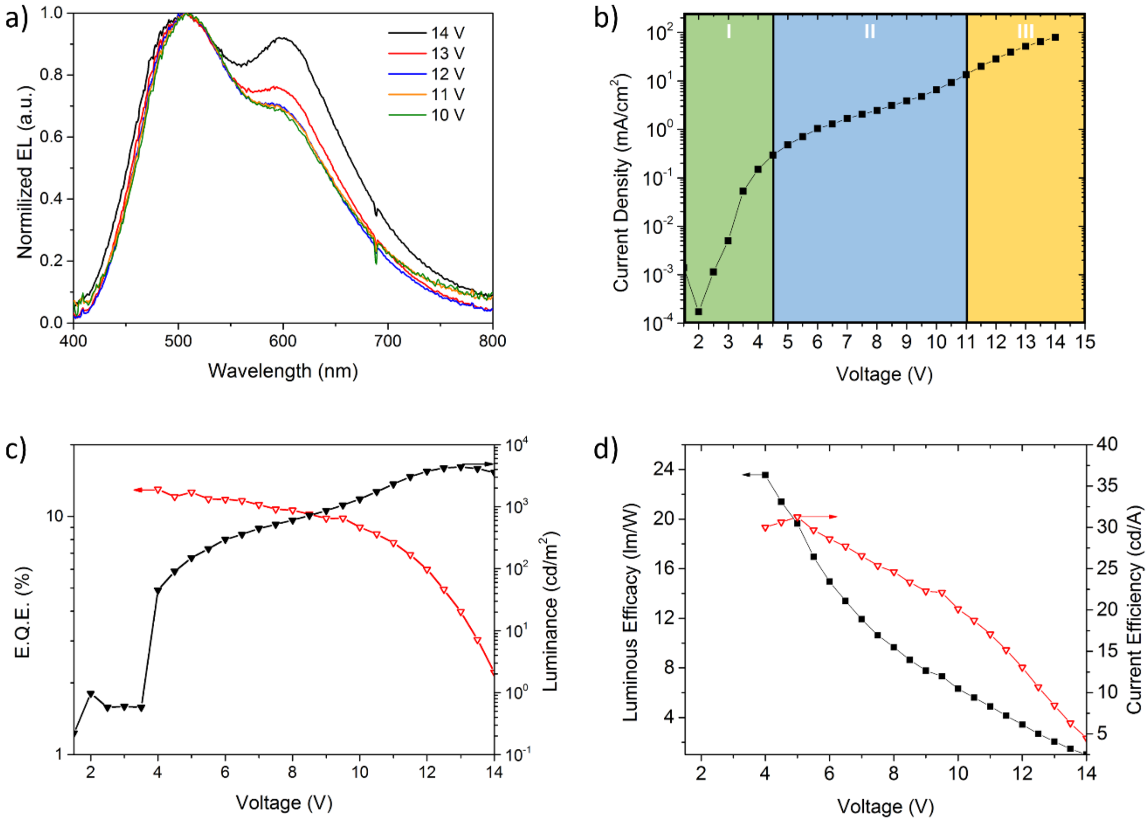

Figure S7. The electrical characterization of the orange green blue structure with thinner layers of DPEPO (7 nm). a) EL spectra at voltages from 10 to 14 V. b) Current density-voltage (JV) with three different current regimes: I the space charge limited current (SCLC); II and III the different carrier balance regimes. c) η_{ext} and luminance dependences with voltage. d) Luminous and current efficiencies of the device.

V_{ON} (V)	L_{MAX} (cd/m^2)	η_{ext}, max (%)	η_L, max (cd/A)	η_P, max (lm/W)	$\eta_{ext}{}^1$ (%)	$\eta_L{}^1$ (cd/A)	$\eta_P{}^1$ (lm/W)	$\eta_{ext}{}^2$ (%)	$\eta_L{}^2$ (cd/A)	$\eta_P{}^2$ (lm/W)
3.5	4372	13.0	31.2	23.6	12.4	31.0	20.5	9.8	22.2	7.4

[1] values taken at 100 cd/m^2 [2] values taken at 1000 cd/m^2

Voltage (V)	CIEx	CIEy	CCT (K)	CRI
10	0.33	0.42	5462	79.8
12	0.33	0.42	5451	80.3
13	0.34	0.41	5282	83.1
14	0.36	0.40	4816	87.5

Table S4. Electrical and optical properties of the OGB devices.

		CCT (K)	CRI
Incandescent	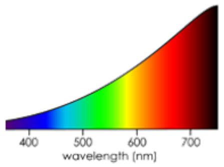	~2500	90-95
Fluorescent	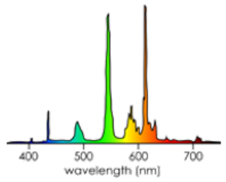	~4000	50-90
Daylight	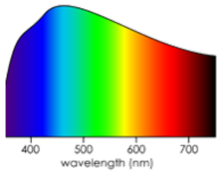	6000	~100
Warm White LED	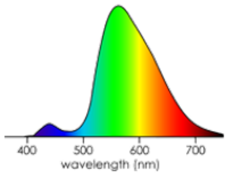	~3000	70
Cool White LED	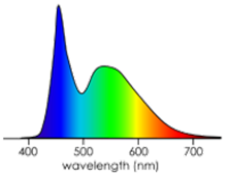	~7000	90

Figure S8. Different light sources and their corresponding Correlated Colour Temperatures and Colour Rendering Index as a means to compare with the devices obtained in this study.[6]

References

1. Dos Santos, P. L., Ward, J. S., Bryce, M. R. & Monkman, A. P. Using Guest-Host Interactions to Optimize the Efficiency of TADF OLEDs. *J. Phys. Chem. Lett.* **7,** 3341–3346 (2016).
2. Dos Santos, P. L. *et al.* Engineering the singlet-triplet energy splitting in a TADF molecule. *J. Mater. Chem. C* **4,** 3815–3824 (2016).
3. Data, P. *et al.* Dibenzo[a,j]phenazine-Cored Donor-Acceptor-Donor Compounds as Green-to-Red/NIR Thermally Activated Delayed Fluorescence Organic Light Emitters. *Angew. Chemie - Int. Ed.* **55,** 5739–5744 (2016).
4. Xu, H. *et al.* Application of chelate phosphine oxide ligand in EuIII complex with mezzo triplet energy level, highly efficient photoluminescent, and electroluminescent performances. *J. Phys. Chem. B* **110,** 3023–3029 (2006).

5. Adachi, C. *et al.* Endothermic energy transfer: A mechanism for generating very efficient high-energy phosphorescent emission in organic materials. *Appl. Phys. Lett.* **79,** 2082–2084 (2001).
6. Floroiu, V. A. The ABCs of truly energy efficient LED lighting. *linkedin* (2015). Available at: https://www.linkedin.com/pulse/abcs-truly-energy-efficient-led-lighting-victor-adrian-floroiu. (Accessed: 18th January 2017)
